# Breast metastasis from medullary thyroid carcinoma mimicking ductal carcinoma with neuroendocrine differentiation

**DOI:** 10.1002/cnr2.1292

**Published:** 2020-10-07

**Authors:** Juan B. Laforga, Eva Dominguez, Francisco Ignacio Aranda

**Affiliations:** ^1^ Department of Pathology Hospital de Denia Denia Spain; ^2^ Department of Radiology Hospital de Denia Denia Spain; ^3^ Department of Pathology Hospital General Universitario de Alicante Alicante Spain

**Keywords:** breast metastasis, core biopsy, diagnosis, immunohistochemistry, medullary thyroid carcinoma

## Abstract

**Background:**

Medullary thyroid carcinoma very rarely metastasizes to the breast. Hematogenous spread to the liver, lungs, or mediastinum is more common.

**Case:**

We describe the morphologic and immunohistochemical features of a 63‐year‐old woman who presented with a BIRADS‐5 category nodule in the right breast and enlarged axillary lymph nodes. Core biopsy showed suggested breast cancer with neuroendocrine or apocrine differentiation. The immunohistochemical profile showed (RE−/RP−/HER‐2−) and Ki67 10%. Chromogranin and synaptophysin were positive; AR and GCDFP‐15 were negative. On reviewing the patient's clinical history, it was discovered that she had been treated for medullary thyroid carcinoma 15 years earlier. Additional stains showed positivity for TTF‐1, CEA, and calcitonin. These findings were consistent with a diagnosis of breast metastasis from medullary thyroid carcinoma. We discuss briefly the morphologic features and the possible key features in order to make an accurate diagnosis.

**Conclusion:**

This case highlights the importance of investigating a history of cancer in patients with discordant or unusual histologic or immunohistochemical findings, as this can help avoid misdiagnosis and inappropriate treatment.

## 1 INTRODUCTION

Medullary thyroid carcinoma (MTC) is a malignant tumor showing parafollicular C‐cell differentiation. It typically secretes calcitonin and accounts for 2% to 4% of all thyroid malignancies.^1^ Ten‐year survival rates range between approximately 45% and 80%, indicating the relatively indolent nature of this tumor.^2^ Seventy percentage of MTCs are sporadic and unilateral, while 30% are hereditary and typically multicentric and bilateral.^3^, ^4^, ^5^ MTC usually metastasizes to lymph nodes in the neck and mediastinum. In patients with advanced disease, it can be found at distant sites such as the lungs, liver, adrenal glands, and bone. Mammary gland metastasis of MTC is extremely rare and may be difficult to differentiate from other tumors. We describe a metastatic MTC in the breast, mimicking a ductal infiltrating carcinoma with neuroendocrine differentiation.

## 2 CASE

**FIGURE 1:**
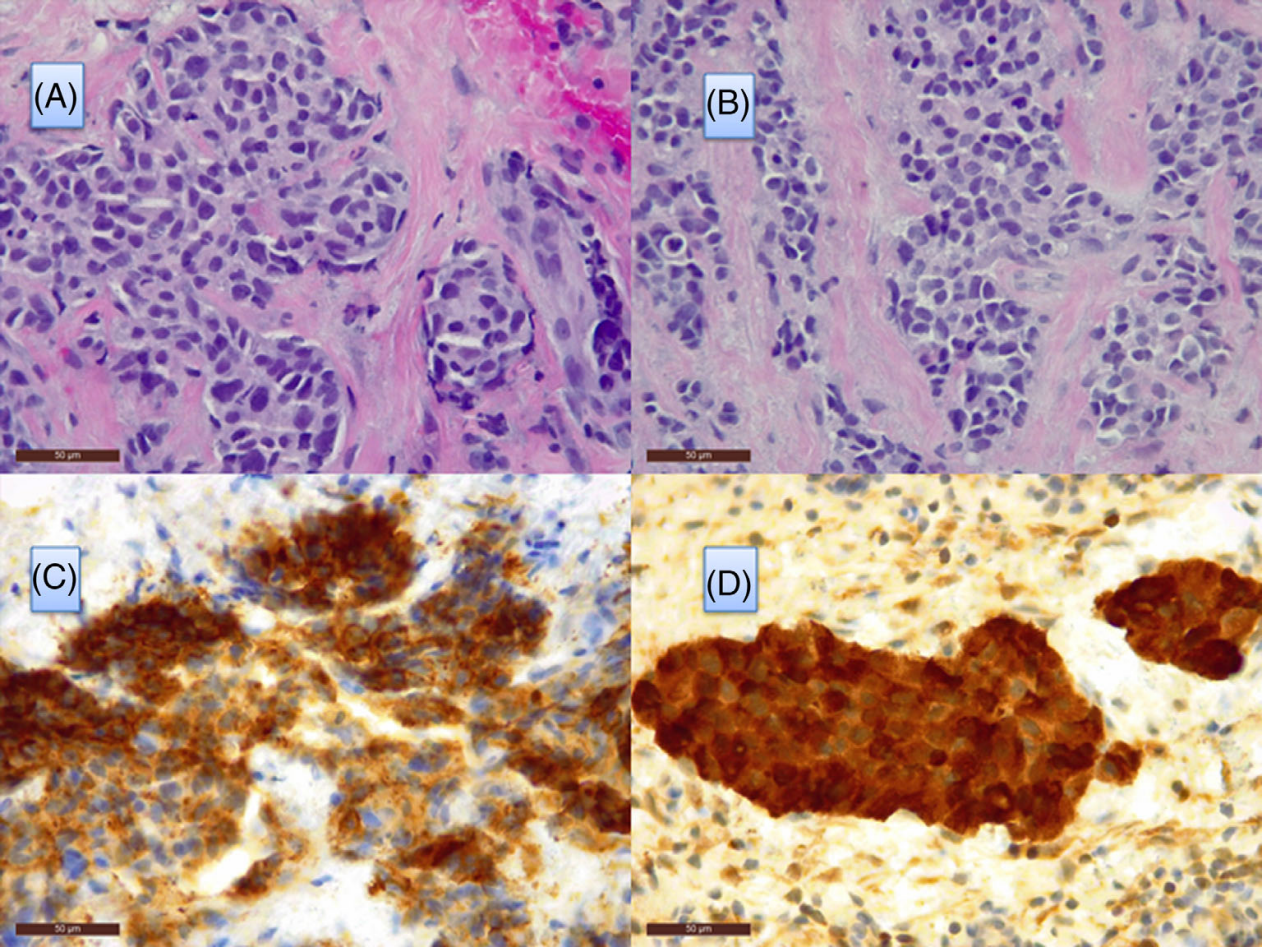
Metastasis from medullary thyroid carcinoma to the breast. A, Lobular cancerization. Tumor invasion showing an organoid pattern formed by cells with moderate nuclear pleomorphism and eosinophilic cytoplasm (hematoxylin‐eosin ×400). B, Collagenous stroma in the breast showing invasion by masses and cords of atypical epithelial cells (hematoxylin‐eosin ×400). C, Tumor cells showing cytoplasmic positivity for CEA (CEA ×400). D, All the cells showed strong, diffuse staining for calcitonin (calcitonin ×400)

**FIGURE 2:**
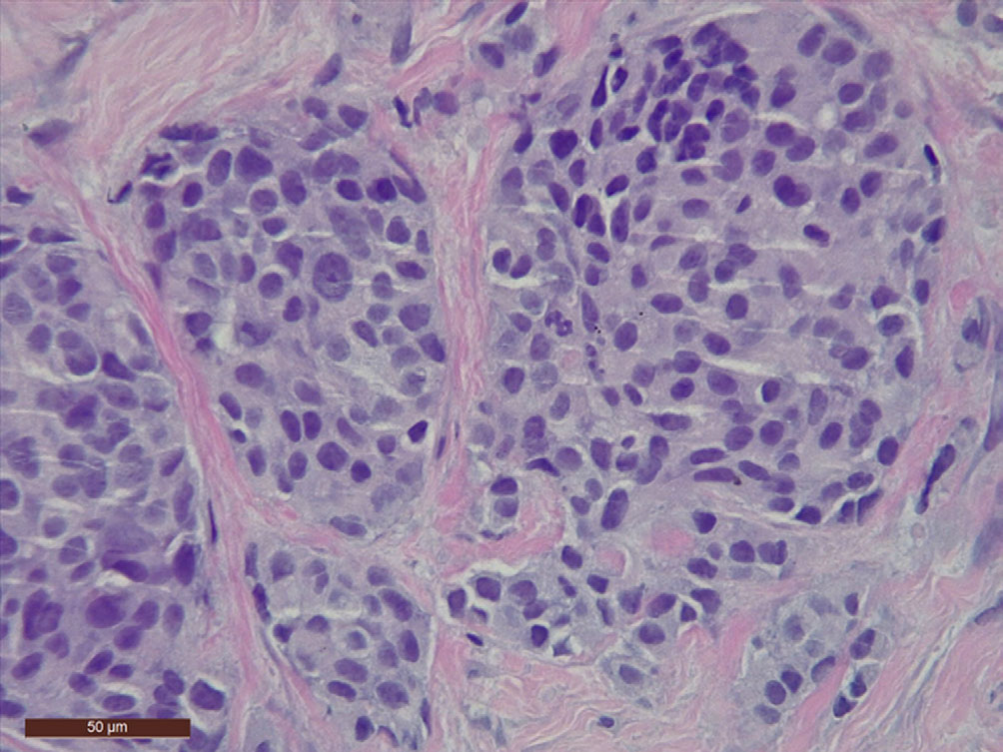
Cells showing round or ovoid nuclei with fine salt‐and‐pepper chromatin and occasional prominent nucleoli (hematoxylin–eosin ×400)

In 2010, a 63‐year‐old woman was seen at Hospital de Denia for a BIRADS‐5 category nodule in the right breast and enlarged axillary lymph nodes. Core biopsy showed an epithelial tumor formed by round, medium‐sized cells with a plasmacytoid morphology in some cases and a spindle shape in others. There was also evidence of lobular cancerization (Figure 1A,B). The cells had central, hyperchromatic, and occasionally displaced nuclei with fine salt‐and‐pepper chromatin and occasional prominent nucleoli. The cytoplasms were abundant and granular (Figure 2). Morphology suggested breast cancer with neuroendocrine or apocrine differentiation. The immunohistochemical study was positive for cadherin and negative for ER, PR, and HER‐2. The Ki67 proliferation index was 10%. Additional studies showed strong cytoplasmic staining for neuroendocrine markers (chromogranin and synaptophysin) and negative results for apocrine markers (androgen receptor and GCDFP‐15). A tentative diagnosis of invasive ductal carcinoma with neuroendocrine differentiation was made. On reviewing the patient's clinical history, it was discovered that she had undergone total thyroidectomy for MTC at another hospital (Hospital General de Alicante) in 1989. She had been treated with multiple anticancer drugs, radiation therapy, and iodine brachytherapy. Metastasis to the liver was detected in 2003. The treatment in this case was sandostatin and tamoxifen. In 2007, a computed tomography (CT) scan of the chest and abdomen showed disease progression with enlarged bilateral cervical lymph nodes. She was treated with dacarbazine (DTIC) and oral fluoropyrimidines. Further progression was observed in 2008. The patient was started on sorafenib for compassionate use, but this was changed to sunitinib (also for compassionate use) because she developed hand‐foot syndrome.

**FIGURE 3:**
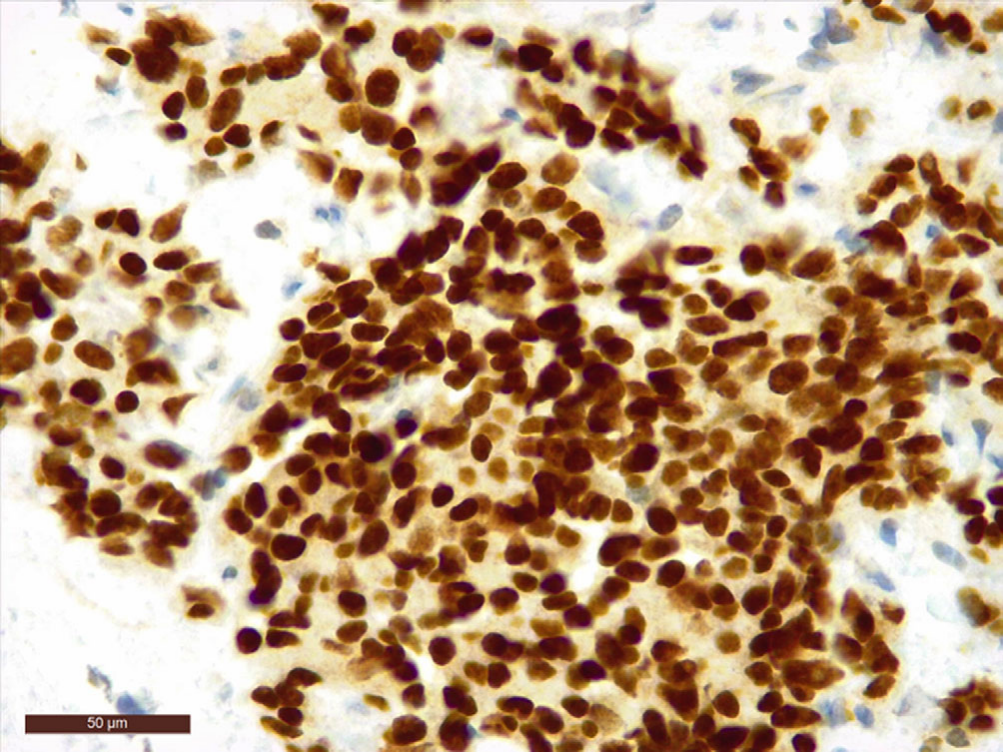
Tumor cells showing strong, diffuse nuclear staining with TTF‐1 (TTF‐1 ×400)

With this information, we performed another immunohistochemical study, which showed positivity for thyroid transcription factor 1 (Figure 3), carcinoembryonic antigen (CEA), and calcitonin (Figure 1C,D). GATA3 stain was negative. These findings were consistent with a diagnosis of breast metastasis from MTC. We had the opportunity to re‐examine the post‐thyroidectomy biopsy sections from the primary MTC. These showed a nest pattern formed by cells with fine nuclear chromatin and abundant granular cytoplasm that stained positively with CEA and calcitonin.

**FIGURE 4:**
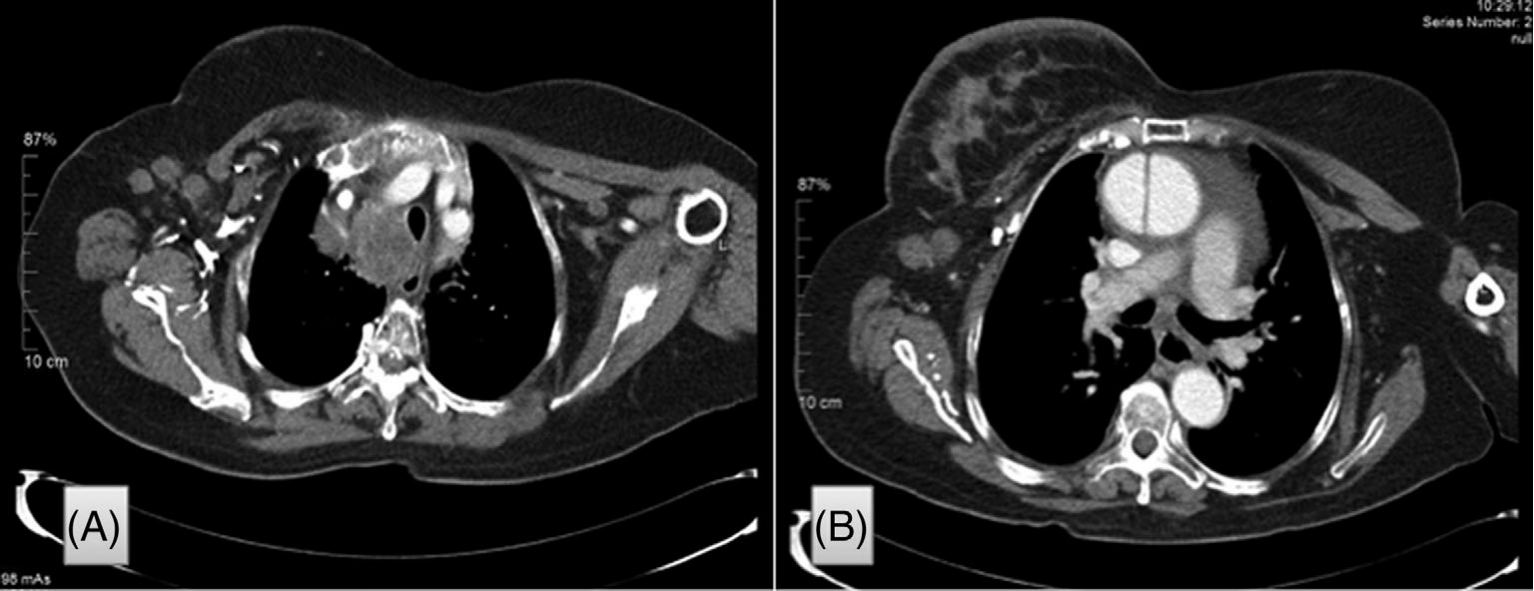
Chest computed tomography scan. A, Enlarged metastatic lymph nodes in the right axillary region. B, Thickened skin on the right breast and enlarged mediastinal lymph nodes

The imaging studies performed at our hospital (CT scan of the chest and abdomen) showed multiple lesions in the liver and enlarged mediastinal and paratracheal lymph nodes displacing the trachea and esophagus, in addition to enlarged lymph nodes in the right axillary region (Figure 4A,B). The scan also showed diffuse thickening of the skin on the right breast. Clinically, the patient had intermittent dysphagia for liquids and poor and deteriorating general health. She was treated with pazopanib within a compassionate use program. She died in February 2012, 23 years after the initial diagnosis.

## 3 DISCUSSION

Breast metastases are uncommon and may be multifocal and/or bilateral.^6^ The most common tumors that cause metastasis to the breast are, in order of frequency, contralateral breast cancer, lung cancer, ovarian cancer, thyroid cancer, and rhabdomyosarcoma. An accurate diagnosis is clinically important, as most patients will have disseminated disease elsewhere (Stage IV). Ultrasound and mammography show nonspecific findings for MTCs that have metastasized to the breast, particularly in the case of solitary tumors. Spiculation and microcalcifications are not seen, unlike in syndromic MTC, where microcalcifications are common. These metastases are therefore difficult to distinguish from primary breast carcinoma.^7^ Mammography may show nodular lesions with diffuse skin thickening,^8^ as in our case. Stage IV disease is typically associated with a poor prognosis and calls for systemic treatment or palliative care. Patient with MTC and distant metastasis can, however, survive for many years, although the 10‐year mortality rate is 65%.^2^ Histologic features of metastasis include a well‐circumscribed lesion with periductal or, as in our case, lobular growth, carcinomatous lymphangitis, and diffuse involvement of the breast parenchyma. These morphological features can simulate invasive lobular breast cancer. Diagnostic clues include absence of carcinoma in situ or flat epithelial atypia, absence of tumor elastosis, and a past history of cancer (although this information is not always available at the time of the histological study, as occurred in our case). MTC very rarely metastasizes to the breast and has only been reported in isolated cases as a late manifestation of already widely disseminated disease.^9^, ^10^, ^11^, ^12^, ^13^ Hematogenous spread to the liver, lungs, or mediastinum is more common. In our case, we initially suspected ductal invasive breast cancer with neuroendocrine differentiation because of both the morphologic features (organoid pattern and cells with a granular eosinophilic cytoplasm with displaced nuclei and fine salt‐and‐pepper chromatin) and the immunohistochemical features (intense staining with neuroendocrine markers). On learning, however, that our patient had a history of MTC, we checked the immunohistochemical expression of calcitonin and CEA and corrected the diagnosis.

This case once again highlights the importance of investigating a history of cancer in patients with discordant or unusual histologic or immunohistochemical findings, as this can help avoid misdiagnosis and inappropriate treatment, such as mastectomy with axillary lymph node dissection or aggressive neoadjuvant treatment for triple‐negative breast cancer.

## ETHICS STATEMENT

Permission to publish this article was given by the patient's family and approved by the ethical committee of our hospital.

## CONFLICT OF INTEREST

The authors have no conflict of interest to be disclosed to this article.

## AUTHOR CONTRIBUTIONS

**Juan Laforga:** Conceptualization; data curation; investigation; resources; writing‐original draft; writing‐review and editing. **Eva Dominguez:** Resources; visualization. **Ignacio Aranda:** Resources; validation; visualization.

## Data Availability

The data that support the findings of this study are available on request from the corresponding author.
